# Supplementary material for: Association of Japanese and Mediterranean Dietary Patterns with Muscle Weakness in Japanese Community-Dwelling Middle-Aged and Older Adults: Post Hoc Cross-Sectional Analysis
Source: Int J Environ Res Public Health. 2022 Oct 3;19(19):12636. doi: 10.3390/ijerph191912636 (PMC9566278; doi:10.3390/ijerph191912636)
Supplement: Supplementary file 1 [file ijerph-19-12636-s001.zip › ijerph-1928551-supplementary.pdf]

Supplemental table S1. Characteristics of sex discrimination in the rJDI12 score quartile range

Characteristics	Male					Female				
	Q1	Q2	Q3	Q4	*P*	Q1	Q2	Q3	Q4	*P*
**Range of scores**	0–4	5–6	7–8	9–12		0–4	5–6	7–8	9–12	
**Participants, n**	746	705	782	565		835	801	892	705	
**Age, years**	61.6 (6.9)	62.3 (6.9)	63.4 (7.0)	64.5 (6.7)	<0.001	61.1 (7.0)	62.3 (7.1)	63.5 (7.0)	64.5 (6.6)	<0.001
**BMI, kg/m2**	23.4 (3.0)	23.6 (3.0)	23.6 (2.8)	23.3 (2.7)	0.432	23.0 (3.5)	22.7 (3.2)	22.9 (3.2)	22.7 (3.1)	0.216
**Cerebrovascular diseases, n (%)**					0.461					0.069
**- Yes**	27 (3.6)	28 (4.0)	24 (3.1)	26 (4.6)		22 (2.6)	12 (1.5)	16 (1.8)	16 (2.3)	
**- No**	476 (63.8)	443 (62.8)	512 (65.5)	378 (66.9)		486 (58.2)	486 (60.7)	570 (63.9)	455 (64.5)	
**- Missing**	243 (32.6)	234 (33.2)	246 (31.5)	161 (28.5)		327 (39.2)	303 (37.8)	306 (34.3)	234 (33.2)	
**Coronary heart diseases, n (%)**					0.123					0.166
**- Yes**	81 (10.9)	65 (9.2)	89 (11.4)	81 (14.3)		66 (7.9)	61 (7.6)	80 (9.0)	56 (7.9)	
**- No**	422 (56.6)	406 (57.6)	447 (57.2)	323 (57.2)		442 (52.9)	437 (54.6)	506 (56.7)	415 (58.9)	
**- Missing**	243 (32.6)	234 (33.2)	246 (31.5)	161 (28.5)		327 (39.2)	303 (37.8)	306 (34.3)	234 (33.2)	
**Diabetes, n (%)**					0.032					0.004
**- Yes**	74 (9.9)	101 (14.3)	104 (13.3)	89 (15.8)		69 (8.3)	61 (7.6)	56 (6.3)	36 (5.1)	
**- No**	429 (57.5)	370 (52.5)	432 (55.2)	315 (55.8)		440 (52.7)	437 (54.6)	530 (59.4)	435 (61.7)	
**- Missing**	243 (32.6)	234 (33.2)	246 (31.5)	161 (28.5)		326 (39.0)	303 (37.8)	306 (34.3)	234 (33.2)	
**Cancer, n (%)**					0.488					0.166
**- Yes**	27 (3.6)	29 (4.1)	36 (4.6)	30 (5.3)		38 (4.6)	39 (4.9)	44 (4.9)	30 (4.3)	
**- No**	476 (63.8)	442 (62.7)	500 (63.9)	374 (66.2)		470 (56.3)	459 (57.3)	542 (60.8)	441 (62.6)	

**- Missing**	243 (32.6)	234 (33.2)	246 (31.5)	161 (28.5)		327 (39.2)	303 (37.8)	306 (34.3)	234 (33.2)	
**Smoking status, n (%)**					<0.001					<0.001
**- Current**	288 (38.6)	253 (35.9)	206 (26.3)	151 (26.7)		110 (13.2)	88 (11.0)	67 (7.5)	35 (5.0)	
**- Former**	297 (39.8)	282 (40.0)	381 (48.7)	279 (49.4)		79 (9.5)	61 (7.6)	58 (6.5)	37 (5.2)	
**- Never**	143 (19.2)	152 (21.6)	178 (22.8)	122 (21.6)		620 (74.3)	632 (78.9)	744 (83.4)	616 (87.4)	
**- Missing**	18 (2.4)	18 (2.6)	17 (2.2)	13 (2.3)		26 (3.1)	20 (2.5)	23 (2.6)	17 (2.4)	
**Alcohol consumption, n (%)**					0.213					0.735
**- Every day**	271 (36.3)	271 (38.4)	305 (39.0)	186 (32.9)		75 (9.0)	63 (7.9)	79 (8.9)	46 (6.5)	
**- Sometimes**	274 (36.7)	264 (37.4)	279 (35.7)	245 (43.4)		264 (31.6)	249 (31.1)	285 (32.0)	231 (32.8)	
**- Never**	186 (24.9)	157 (22.3)	181 (23.1)	121 (21.4)		459 (55.0)	452 (56.4)	485 (54.4)	387 (54.9)	
**- Missing**	15 (2.0)	13 (1.8)	17 (2.2)	13 (2.3)		37 (4.4)	37 (4.6)	43 (4.8)	41 (5.8)	
**Time spent walking, n (%)**					<0.001					<0.001
**- ≥1 h/d**	263 (35.3)	286 (40.6)	357 (45.7)	262 (46.4)		308 (36.9)	332 (41.4)	396 (44.4)	331 (47.0)	
**- 0.5-1 h/d**	258 (34.6)	253 (35.9)	263 (33.6)	193 (34.2)		310 (37.1)	305 (38.1)	338 (37.9)	250 (35.5)	
**- <0.5 h/d**	198 (26.5)	151 (21.4)	141 (18.0)	99 (17.5)		199 (23.8)	155 (19.4)	149 (16.7)	113 (16.0)	
**- Missing**	27 (3.6)	15 (2.1)	21 (2.7)	11 (1.9)		18 (2.2)	9 (1.1)	9 (1.0)	11 (1.6)	
**TMIG-IC score, points**	5 (5–5)	5 (5–5)	5 (5–5)	5 (5–5)	0.164	5 (5–5)	5 (5–5)	5 (5–5)	5 (5–5)	0.036
**Energy, kcal/d**	1584.3 (481.8)	1942.3 (498.5)	2226.2 (556.7)	2615.9 (647.8)	<0.001	1314.3 (374.9)	1633.8 (400.7)	1867.6 (456.6)	2243.3 (571.2)	<0.001

Data are expressed as numbers (percentage) or mean (SD) or median (IQR: 25%, 75%) unless otherwise indicated.
rJDI12, 12-component revised Japanese Diet Index; BMI, body mass index; TMIG, Tokyo Metropolitan Institute of Gerontology Index of Competence.

Supplemental table S2. Characteristics of sex discrimination in the aMed score quartile range

Characteristics	Male					Female				
	Q1	Q2	Q3	Q4	*P*	Q1	Q2	Q3	Q4	*P*
**Range of scores**	0–3	4	5	6–8		0–3	4	5	6–8	
**Participants, n**	1,151	694	583	370		1,555	744	620	314	
**Age, years**	61.7 (6.9)	63.4 (7.0)	63.6 (6.9)	64.3 (6.6)	<0.001	61.6 (7.1)	63.5 (6.9)	63.7 (6.7)	65.3 (6.5)	<0.001
**BMI, kg/m2**	23.3 (3.0)	23.6 (2.8)	23.7 (3.0)	23.4 (2.7)	0.073	22.8 (3.3)	22.8 (3.3)	22.8 (3.2)	23.1 (3.1)	0.498
**Cerebrovascular diseases, n (%)**					0.153					0.141
**- Yes**	40 (3.5)	23 (3.3)	27 (4.6)	15 (4.1)		25 (1.6)	19 (2.6)	14 (2.3)	8 (2.5)	
**- No**	718 (62.4)	452 (65.1)	385 (66.0)	254 (68.6)		933 (60.0)	461 (62.0)	399 (64.4)	204 (65.0)	
**- Missing**	393 (34.1)	219 (31.6)	171 (29.3)	101 (27.3)		597 (38.4)	264 (35.5)	207 (33.4)	102 (32.5)	
**Coronary heart diseases, n (%)**					0.003					0.068
**- Yes**	98 (8.5)	88 (12.7)	80 (13.7)	50 (13.5)		114 (7.3)	59 (7.9)	54 (8.7)	36 (11.5)	
**- No**	660 (57.3)	387 (55.8)	332 (56.9)	219 (59.2)		844 (54.3)	421 (56.6)	359 (57.9)	176 (56.1)	
**- Missing**	393 (34.1)	219 (31.6)	171 (29.3)	101 (27.3)		597 (38.4)	264 (35.5)	207 (33.4)	102 (32.5)	
**Diabetes, n (%)**					0.014					0.157
**- Yes**	123 (10.7)	104 (15.0)	82 (14.1)	59 (15.9)		98 (6.3)	59 (7.9)	45 (7.3)	20 (6.4)	
**- No**	635 (55.2)	371 (53.5)	330 (56.6)	210 (56.8)		860 (55.3)	422 (56.7)	368 (59.4)	192 (61.1)	
**- Missing**	393 (34.1)	219 (31.6)	171 (29.3)	101 (27.3)		597 (38.4)	263 (35.3)	207 (33.4)	102 (32.5)	
**Cancer, n (%)**					0.169					0.036
**- Yes**	47 (4.1)	27 (3.9)	28 (4.8)	20 (5.4)		75 (4.8)	26 (3.5)	38 (6.1)	12 (3.8)	
**- No**	711 (61.8)	448 (64.6)	384 (65.9)	249 (67.3)		883 (56.8)	454 (61.0)	375 (60.5)	200 (63.7)	

**- Missing**	393 (34.1)	219 (31.6)	171 (29.3)	101 (27.3)		597 (38.4)	264 (35.5)	207 (33.4)	102 (32.5)	
**Smoking status, n (%)**					<0.001					<0.001
**- Current**	432 (37.5)	213 (30.7)	163 (28.0)	90 (24.3)		165 (10.6)	69 (9.3)	54 (8.7)	12 (3.8)	
**- Former**	484 (42.1)	308 (44.4)	268 (46.0)	179 (48.4)		136 (8.7)	52 (7.0)	31 (5.0)	16 (5.1)	
**- Never**	208 (18.1)	156 (22.5)	137 (23.5)	94 (25.4)		1,214 (78.1)	600 (80.6)	525 (84.7)	273 (86.9)	
**- Missing**	27 (2.3)	17 (2.4)	15 (2.6)	7 (1.9)		40 (2.6)	23 (3.1)	10 (1.6)	13 (4.1)	
**Alcohol consumption, n (%)**					<0.001					0.433
**- Every day**	430 (37.4)	263 (37.9)	198 (34.0)	142 (38.4)		128 (8.2)	68 (9.1)	40 (6.5)	27 (8.6)	
**- Sometimes**	389 (33.8)	269 (38.8)	244 (41.9)	160 (43.2)		502 (32.3)	220 (29.6)	205 (33.1)	102 (32.5)	
**- Never**	313 (27.2)	147 (21.2)	124 (21.3)	61 (16.5)		853 (54.9)	422 (56.7)	345 (55.6)	163 (51.9)	
**- Missing**	19 (1.7)	15 (2.2)	17 (2.9)	7 (1.9)		72 (4.6)	34 (4.6)	30 (4.8)	22 (7.0)	
**Time spent walking, n (%)**					0.130					0.554
**- ≥1 h/d**	444 (38.6)	297 (42.8)	254 (43.6)	173 (46.8)		635 (40.8)	323 (43.4)	267 (43.1)	142 (45.2)	
**- 0.5-1 h/d**	416 (36.1)	239 (34.4)	200 (34.3)	112 (30.3)		590 (37.9)	266 (35.8)	239 (38.5)	108 (34.4)	
**- <0.5 h/d**	257 (22.3)	144 (20.7)	110 (18.9)	78 (21.1)		307 (19.7)	143 (19.2)	109 (17.6)	57 (18.2)	
**- Missing**	34 (3.0)	14 (2.0)	19 (3.3)	7 (1.9)		23 (1.5)	12 (1.6)	5 (0.8)	7 (2.2)	
**TMIG-IC score, points**	5 (5–5)	5 (5–5)	5 (5–5)	5 (5–5)	0.035	5 (5–5)	5 (5–5)	5 (5–5)	5 (5–5)	0.064
**Energy, kcal/d**	2042.5 (672.2)	2058.9 (686.9)	2065.1 (612.0)	2125.5 (604.7)	0.211	1702.6 (552.1)	1754.4 (563.5)	1820.9 (563.7)	1821.1 (564.3)	<0.001

Data are expressed as numbers (percentage) or mean (SD) or median (IQR: 25%, 75%) unless otherwise indicated.
aMed, Alternate Mediterranean Diet; BMI, body mass index; rJDI12, 12-component revised Japanese Diet Index; TMIG, Tokyo Metropolitan Institute of Gerontology Index of Competence.

Supplemental table S3. Comparison of two dietary patterns and nutrient intakes

Nutrient intakes	Group of rJDI12					Group of aMed				
	Q1	Q2	Q3	Q4	*P*	Q1	Q2	Q3	Q4	*P*
Protein, g/d	61.3 (6.9)	62.3 (7.0)	63.4 (7.0)	64.5 (6.6)	<0.001	68.1 (28.1)	74.4 (30.5)	77.9 (28.8)	81.8 (6.6)	<0.001
Fiber, g/d	8.4 (2.9)	11.9 (3.3)	15.5 (4.5)	19.9 (5.5)	<0.001	11.7 (5.0)	14.2 (5.9)	15.7 (5.8)	16.8 (6.1)	<0.001
Vitamin A, µgRAE/d	513 (468)	685 (389)	915 (604)	1152 (702)	<0.001	701 (537)	834 (617)	904 (645)	952 (615)	<0.001
Vitamin C, mg/d	78 (39)	116 (47)	155 (57)	203 (67)	<0.001	111 (61)	144 (70)	158 (68)	172 (70)	<0.001
Vitamin E, mg/d	5.43 (2.03)	7.30 (2.29)	9.21 (3.00)	11.7 (3.66)	<0.001	7.27 (3.18)	8.52 (3.59)	9.29 (3.61)	9.87 (3.70)	<0.001
Calcium, mg/d	378 (169)	524 (181)	678 (232)	872 (282)	<0.001	541 (258)	619 (290)	657 (279)	710 (297)	<0.001
Iron, mg/d	5.6 (1.9)	7.7 (1.9)	9.7 (2.6)	12.3 (3.3)	<0.001	7.7 (3.2)	9.0 (3.5)	9.7 (3.4)	10.2 (3.5)	<0.001
Potassium, mg/d	1,770 (594)	2,438 (653)	3,139 (875)	3,993 (1,066)	<0.001	2,462 (1020)	2,889 (1172)	3,097 (1117)	3,290 (1,155)	<0.001
Magnesium, mg/d	183 (56)	248 (58)	313 (79)	396 (100)	<0.001	249 (96)	288 (107)	310 (104)	334 (110)	<0.001
Saturated fat, g/d	10.2 (4.6)	12.5 (4.8)	14.6 (5.5)	17.1 (6.3)	<0.001	14.0 (6.2)	13.3 (6.0)	12.9 (5.2)	12.3 (5.0)	<0.001
Sodium, mg/d	3,449 (1,001)	4,318 (1,045)	5,111 (1,225)	6,192 (1,561)	<0.001	4,322 (1,440)	4,793 (1,560)	5,077 (1,542)	5,378 (1,617)	<0.001
Sugar, g/d	10.3 (8.6)	12.0 (9.5)	12.8 (9.5)	15.2 (11.1)	<0.001	14.2 (10.5)	11.9 (9.6)	10.8 (8.4)	9.4 (7.9)	<0.001

Data are expressed as mean (SD).

rJDI12, 12-component revised Japanese Diet Index; aMed, Alternate Mediterranean Diet

Supplemental table S4. Relationship between the constituent nutrients of rJDI12 and muscle weakness

	Group of rJDI12				
	Q1	Q2	Q3	Q4	*P* for trend
Protein	1.000 (reference)	0.879 (0.684–1.130)	0.805 (0.614–1.055)	0.738 (0.533–1.023)	0.058
Fiber	1.000 (reference)	0.907 (0.703–1.170)	0.861 (0.644–1.150)	0.820 (0.570–1.178)	0.265
Vitamin A	1.000 (reference)	0.849 (0.664–1.085)	0.740 (0.576–0.951)	0.647 (0.489–0.855)	0.001
Vitamin C	1.000 (reference)	0.880 (0.684–1.133)	0.807 (0.612–1.064)	0.740 (0.529–1.034)	0.067
Vitamin E	1.000 (reference)	0.860 (0.669–1.105)	0.767 (0.584–1.009)	0.683 (0.490–0.952)	0.019
Calcium	1.000 (reference)	0.855 (0.666–1.098)	0.758 (0.577–0.995)	0.669 (0.481–0.932)	0.014
Iron	1.000 (reference)	0.884 (0.685–1.142)	0.815 (0.611–1.087)	0.752 (0.524–1.078)	0.108
Potassium	1.000 (reference)	0.910 (0.705–1.174)	0.867 (0.650–1.157)	0.830 (0.579–1.190)	0.292
Magnesium	1.000 (reference)	0.916 (0.708–1.184)	0.876 (0.652–1.175)	0.844 (0.581–1.226)	0.353
Saturated fat	1.000 (reference)	0.863 (0.674–1.105)	0.775 (0.600–1.000)	0.694 (0.519–0.928)	0.010
Sodium	1.000 (reference)	0.863 (0.670–1.112)	0.775 (0.588–1.021)	0.694 (0.494–0.974)	0.028
Sugar	1.000 (reference)	0.866 (0.678–1.106)	0.779 (0.610–0.995)	0.700 (0.535–0.915)	0.006

Odds ratios adjusted for age (50–54, 55-59, 60–64, 65–69, or ≥70 y), sex, BMI (<18.5, 18.5–25, ≥25 kg/m^2, or missing), IADL score (TMIG-IC score), time spent walking (≥1, 0.5–1, <0.5 h/d, or missing), alcohol consumption (every day, sometimes, never, or missing), smoking status (current, former, never, or missing), history of diseases (cerebrovascular diseases, coronary heart diseases, diabetes, cancer [yes, no, or missing for each term]).

rJDI 12, 12-component revised Japanese Diet Index

Supplemental table S5. Relationship between the constituent nutrients of aMed and muscle weakness

	Group of aMed				
	Q1	Q2	Q3	Q4	*P* for trend
Protein	1.000 (reference)	0.881 (0.701–1.105)	0.751 (0.583–0.966)	1.059 (0.799–1.403)	0.436
Fiber	1.000 (reference)	0.909 (0.722–1.144)	0.795 (0.614–1.030)	1.131 (0.846–1.511)	0.852
Vitamin A	1.000 (reference)	0.857 (0.683–1.076)	0.723 (0.561–0.930)	1.005 (0.759–1.331)	0.238
Vitamin C	1.000 (reference)	0.897 (0.713–1.129)	0.776 (0.599–1.005)	1.100 (0.823–1.470)	0.681
Vitamin E	1.000 (reference)	0.883 (0.702–1.110)	0.758 (0.586–0.979)	1.066 (0.800–1.420)	0.501
Calcium	1.000 (reference)	0.876 (0.698–1.101)	0.747 (0.580–0.963)	1.053 (0.793–1.397)	0.412
Iron	1.000 (reference)	0.895 (0.711–1.125)	0.771 (0.597–0.996)	1.092 (0.820–1.454)	0.625
Potassium	1.000 (reference)	0.903 (0.718–1.135)	0.783 (0.606–1.011)	1.113 (0.836–1.482)	0.735
Magnesium	1.000 (reference)	0.904 (0.719–1.136)	0.785 (0.608–1.014)	1.124 (0.843–1.499)	0.775
Saturated fat	1.000 (reference)	0.860 (0.686–1.079)	0.724 (0.563–0.932)	1.005 (0.759–1.330)	0.235
Sodium	1.000 (reference)	0.881 (0.701–1.107)	0.754 (0.585–0.972)	1.063 (0.800–1.413)	0.469
Sugar	1.000 (reference)	0.859 (0.685–1.079)	0.726 (0.564–0.934)	1.008 (0.760–1.336)	0.250

Odds ratios adjusted for age (50–54, 55-59, 60–64, 65–69, or ≥70 y), sex, BMI (<18.5, 18.5–25, ≥25 kg/m^2, or missing), IADL score (TMIG-IC score), time spent walking (≥1, 0.5–1, <0.5 h/d, or missing), alcohol consumption (every day, sometimes, never, or missing), smoking status (current, former, never, or missing), history of diseases (cerebrovascular diseases, coronary heart diseases, diabetes, cancer [yes, no, or missing for each term]).

aMed, Alternate Mediterranean Diet

Supplemental table S6. Relationship between the combination of adherence to two dietary patterns and muscle weakness

	Adjusted odds ratio	95% confidence interval	*P*
Model 1			
Both low scores	1.000 (reference)		
High rJDI12 and low aMed score	0.783	0.625–0.982	0.034
Low rJDI12 and high aMed score	0.898	0.650–1.242	0.516
Both high scores	0.703	0.507–0.974	0.021
Model 2			
Low rJDI12 and high aMed score	1.000 (reference)		
High rJDI12 and low aMed score	0.872	0.617–1.232	0.437
Both high scores	0.845	0.595–1.199	0.345
Both low scores	1.113	0.805–1.540	0.516

Adjusted for age (50–54, 55–59, 60–64, 65–69, or ≥70 y) and sex, BMI (<18.5, 18.5–25, ≥25 kg/m2, or missing), IADL score (TMIG-IC score), time spent walking (≥1, 0.5-1, <0.5 h/d, or missing), alcohol consumption (every day, sometimes, never, or missing), smoking status (current, former, never, or missing), history of diseases (cerebrovascular disease, coronary heart disease, diabetes, cancer [yes, no, or missing for each term]).

rJDI12, 12-component revised Japanese Diet Index; aMed, Alternate Mediterranean Diet.
